# The Role of Structural Variants in the Genetic Architecture of Parkinson’s Disease

**DOI:** 10.3390/ijms25094801

**Published:** 2024-04-27

**Authors:** Abigail Miano-Burkhardt, Pilar Alvarez Jerez, Kensuke Daida, Sara Bandres Ciga, Kimberley J. Billingsley

**Affiliations:** 1 Laboratory of Neurogenetics, National Institute on Aging, Bethesda, MD 20892, USA; abbylmiano@gmail.com (A.M.-B.); kensuke.daida@nih.gov (K.D.); 2 Center for Alzheimer’s and Related Dementias, National Institute on Aging, Bethesda, MD 20892, USA; pilar.alvarezjerez@nih.gov (P.A.J.); sara.bandresciga@nih.gov (S.B.C.)

**Keywords:** Parkinson’s disease, genetics, structural variants, long-read sequencing

## Abstract

Parkinson’s disease (PD) significantly impacts millions of individuals worldwide. Although our understanding of the genetic foundations of PD has advanced, a substantial portion of the genetic variation contributing to disease risk remains unknown. Current PD genetic studies have primarily focused on one form of genetic variation, single nucleotide variants (SNVs), while other important forms of genetic variation, such as structural variants (SVs), are mostly ignored due to the complexity of detecting these variants with traditional sequencing methods. Yet, these forms of genetic variation play crucial roles in gene expression and regulation in the human brain and are causative of numerous neurological disorders, including forms of PD. This review aims to provide a comprehensive overview of our current understanding of the involvement of coding and noncoding SVs in the genetic architecture of PD.

## 1. Introduction

### 1.1. Genetics of Parkinson’s Disease

Parkinson’s disease (PD) is the second most common neurodegenerative disorder and the most common movement disorder worldwide. It affected approximately 6.2 million individuals in 2015, with predictions estimating this number to double by 2040 [1]. PD has a known genetic component and can be both monogenic and apparently sporadic in nature. Mendelian forms of disease result from highly penetrant, disease-causing variants. Over twenty associated genes have been identified, although robust replication for the majority of these is missing [2]. On the other hand, sporadic PD is caused by common variants with moderate to low risk that increase disease susceptibility. To date, the majority of sporadic PD genetic studies have used large-scale genome-wide association studies (GWASs) focused on single nucleotide variants (SNVs) [3,4]. Through this work, over one hundred independent risk loci have been associated with apparently sporadic PD. However, these common variants explain around 16–30% of the heritable component of PD, leaving a large portion of the genetics of PD unknown [5,6].

### 1.2. Coding vs. Noncoding DNA

DNA can be categorized as either coding or noncoding. Coding DNA refers to regions of the genome that encode proteins, representing approximately 1.5% of the human genome [7]. In contrast, noncoding regions of the genome comprise the vast majority of DNA and include important regulatory elements and noncoding RNAs.

Causal variants identified in neurodegenerative diseases so far have tended to be located in coding regions of DNA, as these are more likely to have a substantial functional impact and are thus implicated in monogenic forms of disease. However, the majority of common variants identified through GWASs are located in noncoding regions, which cumulatively contribute to disease risk. Despite being located in a noncoding region of the genome, these variants can still influence gene expression, protein function, and other molecular processes, thereby contributing to the risk of developing a disease or influencing a trait.

### 1.3. Structural Variants

**Figure 1:**
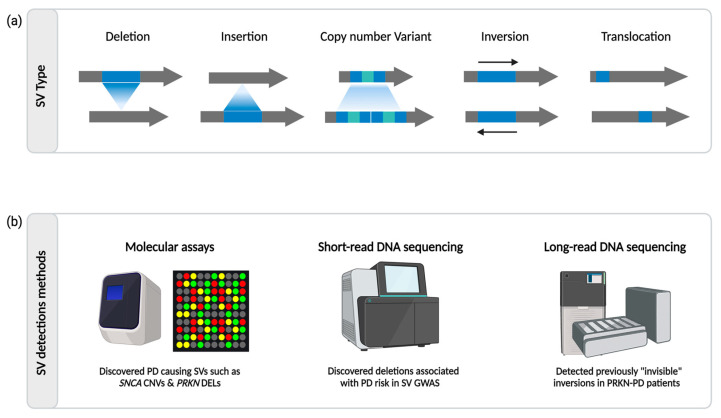
(**a**) Overview of different types of SVs. Each arrow represents a sequence of DNA. The blue portions of the arrows represent a variant. The first row of arrows represents the original DNA sequence while the second row of arrows represents the mutated DNA sequence. (**b**) Overview of the main technologies used to detect SVs. Created with BioRender.com (accessed on 16 January 2024).

The unaccounted “missing heritability” of PD is likely due to the fact that most existing genetic studies have focused on SNVs. Unlike SNVs that result in a single nucleotide change, structural variants (SVs) are any genomic rearrangement including fifty or more nucleotides, and these regions contribute to a more substantial amount of variation in the human genome compared to SNVs [8]. SVs are commonly classified as deletions, duplications, insertions, inversions, and translocations, each portraying distinctive combinations of gains, losses, or rearrangements within the DNA sequence [9,10]. Additionally, SVs include copy number variants (CNVs) and retrotransposon insertions such as *Alu*, LINE-1, and SINE-VNTR-*Alu* (SVA) elements, which can integrate themselves into the human genome. Finally, complex SVs constitute intricate combinations of these events. These genomic variations can considerably influence phenotypes by disrupting gene function, regulation, or altering gene dosage. Recent studies demonstrate that SVs drive functional changes not only across diverse populations but also within various cell and tissue types [10,11] (Figure 1a).

### 1.4. Methods for Detecting Structural Variants

**Figure 2:**
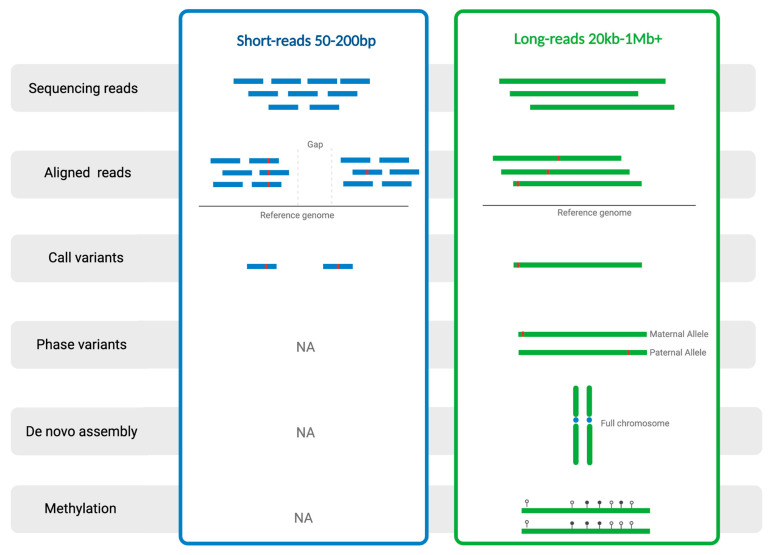
Comparison of the capabilities of short- and long-read sequencing technologies. The left panel displays short blue lines representing reads from short-read sequencing, and the right panel displays long green lines representing reads from long-read sequencing (except for the two vertical green lines which represent a homologous chromosome). The red lines on some of the reads are variants. Adapted from Kono and Arakawa [15]. Created with BioRender.com (accessed on 16 January 2024).

There are several methods for detecting SVs, including molecular assays, short-read DNA sequencing, and, more recently, long-read DNA sequencing (Figure 1b). Many of the first PD SV genetic studies used molecular techniques, such as quantitative polymerase chain reaction (qPCR), multiplex ligation-dependent amplification (MLPA), fluorescent in situ hybridization (FISH), and other microarrays to detect gene dosage. qPCR, in comparison to standard PCR, uses fluorescent labeling to measure the amount of PCR product in real time, which allows for more accurate quantification. MLPA is a variation of a multiplex PCR, which uses probes rather than primers to detect a target sequence and amplifies ligated probes, which can then be quantified using capillary electrophoresis. FISH uses fluorescent probes to precisely localize a specific DNA sequence in an individual cell. However, these methods cannot provide sequence information. Short-read sequencing is a widely used technique in genomics that involves sequencing DNA fragments of relatively small length (typically ranging from 50 to 300 nucleotides). This approach, employed in technologies such as Illumina sequencing, is known for its high throughput and cost-effectiveness. In recent years, hundreds of tools have been developed for detecting SVs from short-read sequencing data, and using these tools, several groups have been successful in identifying SVs associated with disease risk [12,13,14]. However, short-read sequencing faces challenges in accurately calling SVs due to limitations in read length, difficulty in spanning large-scale variations, issues with repetitive regions, and potential chimeric read alignment, which often lead to spurious associations that lack replication. In comparison, “third generation” long-read sequencing technology makes it possible to sequence much larger stretches of DNA. Technologies such as Pacific Biosciences (PacBio) and Oxford Nanopore Technologies (ONT) generate reads spanning tens of thousands to millions of base pairs. Because of this, long-read sequencing offers advantages in resolving complex genetic variation, including repetitive regions and SVs. This technology is particularly valuable for de novo genome assembly, accurately identifying large-scale SVs, and providing a more comprehensive view of the genome’s architecture. Figure 2 gives a detailed overview of the different capabilities of short- and long-read DNA sequencing technologies.

### 1.5. Structural Variants in Neurological Conditions

SVs are well-established drivers of gene regulation in the human brain. In a recent study, Han and colleagues integrated SV calls from nearly fifteen thousand individuals with short-read bulk RNA-sequencing datasets from human post-mortem brain tissue. Through this work, they identified many coding SVs that overlapped with gene expression outliers [11]. Additionally, the authors of a large-scale study performed SV quantitative trait locus (QTL) analyses, and they reported that more than three thousand SVs impact histone modifications, mRNA expression, mRNA splicing, and protein abundance in brain-based short-read datasets [16]. Due to their potential to exert profound phenotypic influences in the brain, numerous mostly coding SVs have been linked with neurological conditions [13] and neurodevelopmental/neuropsychiatric disorders [17]. For example, Phelan-McDermid, or 22q13 deletion syndrome, which most commonly results in intellectual disability (ID), developmental delay, delayed or absent speech, and hypotonia, all of varying severity, can be caused by a deletion at the *SHANK3* gene [18]. Syndromic X-linked ID Lubs type is caused by a duplication of the gene *MECP2,* which almost exclusively occurs in males and is characterized by ID, developmental delay, recurrent infections, delayed speech and motor, hypotonia, seizures, and gastrointestinal dysfunction [19]. Further, Duchenne Muscular Dystrophy can be caused by deletions or duplications in the *DMD* gene [20], and deletions at the *SPAST* gene lead to hereditary spastic paraplegia [21]. In addition to coding SVs being causative of monogenic conditions, there are several SVs that significantly increase an individual’s risk of disease. One example of this is a deletion at 1q21.1, which is associated with an increased risk of autism spectrum disorder (ASD), attention deficit hyperactivity disorder (ADHD), schizophrenia, ID, epilepsy [22], and parkinsonism [23].

In the context of SVs and their role in various forms of parkinsonism, it is noteworthy that a rare SV is associated with X-linked Dystonia Parkinsonism (XDP). XDP, also known as Lubag syndrome, is a rare genetic movement disorder characterized by progressive dystonia and parkinsonism, endemic to the island of Panay in the Philippines [24]. Specifically, XDP is associated with an insertion of an SVA retrotransposon that is inserted into intron 32 of the *TAF1* gene [25,26,27]. This SVA contains a hexameric DNA repeat domain (CCCTCT)n that is variable in length across patients, and the length of this expansion inversely correlates with age at onset (AAO). To understand the functional impact of the SVA, Aneichyk and colleagues demonstrated in cell models that this variant altered *TAF1* splicing and decreased expression of the full-length transcript [28]. Additionally, using induced pluripotent stem cell (iPSC) lines derived from XDP patients, Rakovic and colleagues showed that removal of the SVA using genome editing can successfully rescue *TAF1* expression [29], highlighting the causative nature of the SVA.

This review highlights key studies that have identified SVs associated with monogenic forms of PD (detailed in Supplementary Table S1), which are mostly in coding regions of the genome. We also outline SVs associated with the risk of apparently sporadic PD and finally detail ongoing research efforts to comprehensively catalog SVs in PD through large-scale long-read sequencing initiatives. Notably, while several studies have also reported repeat expansions that are associated with PD, these will not be discussed in the present review as our focus is on SVs.

## 2. Monogenic Parkinson’s Disease

### 2.1. Autosomal Dominant Parkinson’s Disease

#### SNCA/PARK1

The *SNCA* gene, Synuclein Alpha, is located on chromosome 4 (chr4:89724099–89837161) and encodes presynaptic neuronal protein alpha-synuclein [30,31,32]. A build-up of this protein in the brain is the major constituent of Lewy bodies, the main pathological hallmark of synucleinopathies, including PD. Although PD was originally believed to be caused only by environmental factors, in 1997, a missense SNV in *SNCA* was associated with PD [33]. Other point mutations at this locus have also been identified to cause familial PD [34,35,36,37]. However, the first time an SV was associated with *SNCA* was in 2003, when a deleterious triplication of the entire gene was found in a family with autosomal dominant PD [38]. Following this, further studies have continued to identify *SNCA* multiplications (CNVs) as a rare cause of familial parkinsonism [39,40,41,42,43,44,45,46]. In addition to multiple *SNCA* triplication studies in individuals of European ancestry, other studies have reported this multiplication in other populations, such as the Japanese [44,46], Korean [39], South African, and Tunisian [31] populations.

Multiplications of *SNCA* include triplications and duplications. A heterozygous triplication has three copies of the *SNCA* gene on one allele and a single copy of the gene on the other allele, meaning a triplication carrier has a total of four copies of *SNCA*. A heterozygous duplication carrier has two copies of the *SNCA* gene on one allele and one copy on the other, for a total of three copies. Duplication carriers can also be homozygous, meaning they have two copies of *SNCA* on each allele for a total of four copies. In a large family study that comprised 264 families with typical autosomal dominant PD and 22 families with atypical autosomal dominant parkinsonism, about 1.5% of families with typical autosomal dominant PD had an *SNCA* duplication, while 4.5% of atypical families had a triplication [47]. In the typical PD population, duplications are more frequent than triplications. Most multiplications of this locus involve more than one gene and vary widely in length. Differences in multiplication length between families indicate that SV events occurred independently [31]. The rise of these de novo variations may be due to flanking repeat regions, which could cause genomic instability in this region and lead to gaps and breaks [41].

Along with being disease-causing, SVs at the *SNCA* gene are disease-modifying as the *SNCA* copy number determines the AAO and severity of symptoms, indicating a gene dosage effect. These multiplications are not only limited to *SNCA* but can include any number of neighboring genes, from two or three to dozens. However, neither the number of genes nor the size of the multiplication seems to have an effect on disease severity. The phenotype for duplication carriers includes symptoms similar to that of typical PD patients. These symptoms include mild to moderate common parkinsonism motor symptoms, such as postural instability and resting tremor, with little to no cognitive or autonomic dysfunction. However, some individuals have an *SNCA* duplication but no clinical presentation [44], while others show symptoms similar to that of atypical parkinsonian syndromes [39]. It is unclear why this phenotypic heterogeneity occurs, though it could be linked to other genetic modifiers. The phenotype for patients carrying the triplication, along with homozygous duplication carriers, however, includes atypical features reminiscent of dementia with Lewy bodies (DLB). *SNCA* triplication causes early AAO (younger than 50 years) with rapidly evolving, aggressive symptoms, both motor and non-motor, suggesting complete penetrance. Compared to heterozygous duplication carriers, triplication carriers’ motor symptoms are those typically associated with PD, while non-motor symptoms include cognitive impairment and dysautonomia [40]. *SNCA* triplication can also cause levodopa-induced dyskinesia.

A number of studies have looked into the functional effects of these multiplications, particularly the triplication, in vitro and post-mortem. iPSCs derived from patients with an *SNCA* triplication recapitulate the accumulation of alpha-synuclein and doubling of gene expression [48] as seen in post-mortem blood and brain samples [49]. Various pathophysiological mechanisms have been reported to be triggered by overexpression of *SNCA* in mature iPSC-derived dopaminergic cells, such as elevated oxidative stress [50] and mitochondrial [51] and lysosomal dysfunction [52]. Studies have indicated that the *SNCA* locus triplication can impair neuronal differentiation and maturation in PD [53]. Severe neuronal degeneration in the substantia nigra, locus ceruleus, basal nucleus of Meynert, and hippocampal cornu ammonis has been seen in both triplication [40] and duplication carriers [54].

### 2.2. Autosomal Recessive Parkinson’s Disease

#### 2.2.1. PRKN/PARK2

A few years before the *SNCA* triplication was discovered, the Parkin RBR E3 ubiquitin-protein ligase (*PRKN*) gene was first characterized in 1998 when Kitada and colleagues [55] investigated a very large gene consisting of 1.3 Mb and 12 exons on chromosome 6 [chr6:161347417–162727766] [32]. This study identified a Japanese patient with autosomal recessive juvenile parkinsonism who was missing five exons (exons 3–7). This same study also identified an exon 4 deletion in four separate autosomal recessive PD families.

Parkin is an E3 ubiquitin ligase responsible for catalyzing the ubiquitination process and thus the degradation of aberrant proteins. Studies using fibroblasts from *PRKN* SV carriers implicate a loss of ubiquitin ligase function, ultimately leading to the pathogenesis of PD. Proteins are no longer effectively degraded by the parkin-dependent ubiquitin proteasome pathway, which leads to a protein build-up in substantia nigra pars compacta neurons [56]. *PRKN* SV patient fibroblasts [57,58] and mouse models [59,60,61] have been used to exhibit *PRKN* loss-of-function effects such as mitochondrial dysfunction and oxidative stress. Neuropathologically, *PRKN*-linked PD cases show significant substantia nigral degeneration and generally lack Lewy bodies [62,63,64,65,66]. Cases absent of Lewy bodies may be due to a younger AAO.

Causative SVs in *PRKN* have been reported in several other disorders [67], such as ADHD [68], cancer [69], and ASD [70,71]. *PRKN* variants are the most common cause of autosomal recessive PD, affecting 12.5% of individuals (who are either homozygous or compound heterozygous) in this population, and 42.2% of *PRKN* mutation carriers have an AAO less than 21 years [72]. SVs in this region account for 43.2% of *PRKN* mutations in autosomal recessive PD [73].

*PRKN* is located in FRA6E, a frequently observed common fragile site in the human genome, making it susceptible to gaps and breaks [74]. Deletions are the most common SVs among *PRKN* mutations. These deletions can encompass multiple exons or just one. The majority of exonic deletions span the noncoding region of *PRKN* between exons 2 and 4, and within this exonic region, patients most frequently carry deletions of exon 3 [73,75,76]. Patients with *PRKN* mutations on both alleles present with typical early-onset parkinsonism, including slow disease progression, positive levodopa responsiveness, absence of cognitive decline, resting tremor, rigidity, and levodopa-induced dyskinesia. Duplications, triplications, and occasionally inversions have also been reported as a cause of *PRKN* autosomal recessive PD [77].

There has been controversy among *PRKN* pathogenicity in heterozygous patients. Although homozygous and compound heterozygous carriers show a strong association with PD, researchers have also investigated heterozygous carriers with debate among the findings. In some studies, *PRKN* monoallelic mutations were indicated to be disease-causing [78]. However, evidence shows that these heterozygous participants were incorrectly genotyped due to researchers missing second *PRKN* mutations. In a pair of monozygotic twins presenting with early-onset PD, a very large, novel inversion spanning *PRKN* exons 1–11 was identified using long-read sequencing and went undetected using both short-read whole exome sequencing and MLPA [79]. After validating genotyping methods, heterozygous carriers were not found to be at higher risk for PD compared to those without a *PRKN* mutation [76,77]. Single *PRKN* mutations are relatively common in the general population at around 1.8%.

#### 2.2.2. PARK7/DJ-1

Following the discovery of the deletion at *PRKN* was the identification of an SV at Parkinson’s disease gene 7 (*PARK7*), formerly known as *DJ-1*, located on chromosome 1 [chr1:7961711–7985505] [32]. In 2003, *PARK7* was first characterized when a 14 kb homozygous deletion of exons 1–5 was identified in a Dutch family with early-onset parkinsonism [80]. Mutations in *PARK7*, encoding the deglycase protein DJ-1, are a rare cause of familial autosomal recessive PD, affecting less than 2% of cases [81,82]. Although the majority of pathogenic variants in this gene are attributed to missense/nonsense mutations [73], exonic rearrangements have been identified, including a homozygous deletion of exon 5 [83] and another homozygous deletion of *PARK7* exons 1–5. This homozygous deletion of exons 1–5 was present with a second homozygous deletion of exons 1–6 in the neighboring gene *TNFRSF9* [84]. Individuals with homozygous SVs at *PARK7* tend to present with early-onset (20s and 30s) PD with typical features and a suitable levodopa response. A loss-of-function effect is observed in the same Dutch family with the homozygous 1–5 exonic deletion [80], but studies have yet to investigate the downstream effects of these large SVs in *PARK7*. The function of *PARK7* is hypothesized to play a protective role against oxidative stress and neuroinflammation, but the exact mechanism by which it performs this is still debated [85].

#### 2.2.3. PINK1/PARK6

The next SV-harboring gene discovered in autosomal recessive PD was P-TEN-induced putative kinase 1 (*PINK1)*. *PINK1* was first associated with PD in 2004 when Valente and colleagues [86] identified a nonsense and a missense variant located on chromosome 1 [chr1:20633458–20651511] in three consanguineous parkinsonian families [32]. Similar to *PARK7*, *PINK1* mutations are primarily missense [73,87], but SVs have additionally been identified at this locus. SVs include homozygous deletions of exons 6–8 [88], a compound heterozygous deletion of the entire *PINK1* gene [89], homozygous deletions of exons 4–8 [90], homozygous deletions of exons 2–4, homozygous deletions of exon 4, and homozygous deletions of exon 5 [83]. *PINK1* SV carriers tend to have a younger AAO but largely resemble typical idiopathic PD patients with features including a suitable levodopa response and a lack of dementia. *PINK1* phosphorylates parkin and ubiquitin to degrade damaged mitochondria [91]. Although mouse models have yet to accurately mirror the neurodegeneration seen in PD patient brains, more recent studies in monkeys with *PINK1* deletions show deterioration of the cortex, striatum, and substantia nigra [92,93].

## 3. Apparently Sporadic Parkinson’s Disease

### 22Q11.2 Deletion

In addition to SVs being implicated as causative of monogenic forms of PD, numerous instances highlight SVs that are also genetic risk factors. One such region is located on chromosome 22q11.2, a segment in the human genome prone to meiotic chromosome rearrangements. These rearrangements can result in deletions, contributing to a spectrum of heterogeneous disorders. Among these, 22q11.2 deletion syndrome (22q11.2DS), historically known as DiGeorge syndrome, is the most common and well characterized of these disorders, occurring in ~1 in every 4000 live births [94,95,96,97]. The deletion can span 1.5–3 Mb and primarily arises de novo, although 5% of the events are inherited and the clinical phenotype varies widely. These include medical features such as congenital heart disease, autoimmune disease, immunodeficiency, palatal abnormalities, developmental delays, and learning difficulties [98].

Several studies have established a link between 22q11.2DS and increased risk of PD. Early independent case reports documented the co-occurrence of early-onset parkinsonism and 22q11.2DS [99,100]. Further, Butcher and colleagues reported that in a group of 68 young adults (18–34 years) with the syndrome, four had been diagnosed with early-onset PD [101]. The authors concluded that individuals with 22q11.2DS are estimated to have at least a twenty times increased risk of PD compared with estimates for the general population. Expanding on this association, in 2016, Mok and colleagues conducted an extensive analysis using SNV array datasets [102]. Their large-scale investigation of 22q11.2 deletions involved 9387 cases with apparently sporadic PD and 13,863 controls from European ancestry. Through this work, they identified that chromosome 22q11.2 deletions were enriched in PD cases (8 carriers in PD cases and none in controls). Moreover, PD patients carrying a deletion had an earlier AAO compared to non-carriers (mean 42.1 years vs. mean 61 years, respectively).

## 4. Structural Variants as PD Genetic Risk Factors

Large-scale GWASs have enabled well-powered and unbiased analyses for identifying genetic risk factors. These genetic studies have played a fundamental role in discovering the PD risk loci known today. But, traditionally, due to the instability of accurately genotyping SVs at scale, these studies only focused on SNVs. Recent advances in short-read sequencing technologies and SV detection tools now allow for assessing the contribution of SVs to disease risk in large cohorts. Through this, large-scale SV GWASs have begun to identify SV genetic risk factors associated with various neurodegenerative diseases, including amyotrophic lateral sclerosis [103], Alzheimer’s disease [104], non-Alzheimer’s dementias (Lewy body dementia and frontotemporal dementia) [13], and PD [12].

In 2023, the first PD SV GWAS leveraged the SV detection tool GATK-SV [105] and called SVs using short-read sequencing whole-genome data from 2585 PD cases and 2779 controls from European ancestry. GATK-SV is a “gold standard” SV discovery pipeline developed by the Broad Institute that runs five different SV tools to capture all types of SVs. Using machine learning algorithms, it then merges together and filters the SV calls and outputs one final group vcf file that can be used for downstream genetic analyses [106]. Overall, after the implementation of this pipeline in the PD dataset, over 200,000 SVs were identified, and a mean of ~5600 SVs were detected per genome. Given that short-read sequencing data can still yield many false positives, especially in repetitive regions, it is crucial to validate all SVs of interest with additional molecular assays such as PCR or long-read DNA sequencing [107]. After generating high-quality ONT long-read sequencing from a subset of matched samples, each genome carried a mean of ~27,000 SVs overall. Through ONT validation, three deletions were confirmed that tagged known PD SNVs. Despite identifying these new SVs associated with PD risk, further benchmarking studies comparing the short- and long-read sequencing data demonstrated that ~30% of the SVs detected using short-read sequencing methods were false positives, and ~85% of the SVs detected by the long-read sequencing data were missed by the short-read caller [12]. This highlights that, although analysis of SVs using short-read sequencing datasets can yield valuable insights, it lacks the power to accurately detect most of the SVs in the human genome.

## 5. Future Directions

To elucidate the genetic architecture of PD on a global scale, it is crucial to assess the impact of genetic variation across large, diverse populations. To date, however, the majority of PD GWASs have only been based on European samples. In fact, a recent systematic review quantifying the non-European representation in existing PD GWAS efforts revealed that, despite comprising only 16% of the global population, ~62% of all participants in PD genetic studies were European. This disparity was even more pronounced for neurodegenerative diseases as a whole, with only ~20% of participants in these GWASs being of non-European ancestry. Although there are several consortia-based efforts to improve diversity in PD research, much more is needed to better understand the risk of PD in other populations [108].

Gaining a comprehensive understanding of PD genetics, as outlined throughout this review, also requires the analysis of all forms of genetic variation, extending beyond just SNVs. Although most of the new efforts in the PD space are still focused on generating SNV datasets, these datasets can still be leveraged in conjunction with new SV reference panels to impute SVs into large datasets to gain valuable insights into the role of SVs. Additionally, there are several large-scale long-read sequencing efforts that aim to catalog SVs across diverse populations, including NIH-led initiatives such as the All of Us (AOU) initiative [109] and the Center of Alzheimer’s Disease and Related Dementias (CARD) long-read sequencing initiative [110,111]. Most importantly, in terms of PD-specific initiatives, the Global Parkinson’s Genetics Program (GP2) is conducting a large-scale ONT long-read initiative, which is split broadly into two sections [112,113]. The first section is part of the “Monogenic Network”, whereby samples that have been prioritized based on AAO, family structure, and negative results from short-read sequencing will undergo long-read sequencing to identify new causal variants. Additionally, ~1000 samples will be sequenced from a cohort of apparently sporadic PD patients and controls with the aim of identifying new genetic risk factors and resolving existing known risk loci.

Finally, another promising avenue for future research involves studying the impact of somatic SVs in PD. While the present review has focused on germline SVs, which are genetic variations that can be inherited from parent to offspring, somatic variation is a genetic variation that occurs post-zygotically and can consequently lead to mosaicism in the human brain. However, detecting somatic variation accurately is challenging and often requires high-depth sequencing to reliably identify variants present in a small subset of cells. Despite these difficulties, recent studies have started to address the role of somatic SVs in PD. Mokreter and colleagues detected somatic SVs in *SNCA* in multiple synucleinopathies, including PD and multiple system atrophy (MSA) [114], and using single-cell whole genome sequencing (WGS), Perez-Rodriguez and colleagues report that MSA patients have large CNVs in approximately 30% of all cells [115]. But, these studies have been limited by current technology, which has prevented them from accurately determining the breakpoints of somatic SVs, analyzing a large number of samples, and detecting CNVs smaller than 1 Mb in size. Moving forward, advancements in tools combining high-throughput long-read sequencing and single-cell methods to overcome previous limitations hold the potential to offer a more comprehensive understanding of the impact of somatic SVs in PD in the future.

### Conclusions

While many SVs are known to be associated with PD, our understanding of the overall role that SVs play in PD genetics remains very incomplete. This review highlights that conventional techniques frequently miss potential disease-relevant SVs, as exemplified by the recent identification of a large, rare *PRKN* inversion observed in Japanese PD patients. Long-read sequencing emerges as a powerful tool for resolving these complicated regions of the genome, making ongoing research initiatives that leverage long-read sequencing on a population scale particularly promising. The insights gained from these initiatives are anticipated to revolutionize our understanding of the genetic basis of PD, ultimately facilitating the development of more targeted and effective therapeutic interventions.

## Data Availability

Not applicable.

## Supplementary Materials

The supporting information can be downloaded at: https://www.mdpi.com/article/10.3390/ijms25094801/s1.

## Abbreviations

22q11.2DS	22q11.2 deletion syndrome
AAO	Age at onset
aCGH	Array comparative genomic hybridization
ADHD	Attention-deficit hyperactivity disorder
ASD	Autism spectrum disorder
AOU	All of Us
CARD	Center for Alzheimer’s and Related Dementias
CNV	Copy number variant
DaTSCAN	Dopamine transporter scan
DHPLC	Denaturing high performance liquid chromatography
DLB	Dementia with Lewy bodies
FISH	Fluorescent in situ hybridization
GP2	Global Parkinson’s Genetics Program
GWAS	Genome-wide association study
ID	Intellectual disability
iPSC	Induced pluripotent stem cell
MRI	Magnetic resonance imaging
MLPA	Multiplex ligation-dependent amplification
MSA	Multiple system atrophy
NGS	Next-generation sequencing
ONT	Oxford Nanopore Technologies
*PARK*	Parkinson’s disease gene
PD	Parkinson’s disease
PET	Positron emission tomography
*PINK1*	P-TEN-induced putative kinase 1
*PRKN*	Parkin RBR E3 ubiquitin-protein ligase
qPCR	Quantitative polymerase chain reaction
QTL	Quantitative trait locus
*SNCA*	Synuclein alpha
SPECT	Single-photon emission computed tomography
SNV	Single nucleotide variant
SV	Structural variant
SVA	SINE-VNTR-*Alu*
WGS	Whole genome sequencing
XDP	X-linked Dystonia Parkinsonism
