# Supplementary material for: The Role of Structural Variants in the Genetic Architecture of Parkinson’s Disease
Source: Int J Mol Sci. 2024 Apr 27;25(9):4801. doi: 10.3390/ijms25094801 (PMC11084710; doi:10.3390/ijms25094801)
Supplement: Supplementary file 1 [file ijms-25-04801-s001.zip › ijms-2948957-supplementary.pdf]

Gene	Type of Structural Variant	Method of Detection	Functional Effects	References
SNCA	CNV: Triplication	qPCR (detected), FISH (confirmed)	NA	Singleton et al., 2003 PMID: 14593171
	CNV: Triplication	Semiquantitative multiplex PCR (detected)	Doubling of SNCA expression (gene dosage increase), severe neuronal loss in hippocampal CA2/3, in the substantia nigra and locus coeruleus, and in the basal nucleus of Meynert	Farrer et al., 2004 PMID: 14755720
	CNV: Triplication, Duplication	qPCR (detected), microsatellite marker analysis (confirmed)	NA	Fuchs et al., 2007 PMID: 17251522
	CNV: Triplication, Duplication	Semiquantitative multiplex PCR (detected), Affymetrix 250k SNP microarrays (determined size)	NA	Ibáñez et al., 2009 PMID: 19139307
	CNV: Triplication	Semiquantitative multiplex PCR (detected), MLPA (confirmed)	NA	Sekine et al., 2010 PMID: 20818659
	CNV: Triplication	MLPA (detected), qPCR (confirmed)	NA	Keyser et al., 2010 PMID: 20013014
	CNV: Triplication	MLPA (detected), Illumina HumanOmniExpress BeadChip array (confirmed copy number gain), FISH (confirmed)	Frontoparietal atrophy using brain magnetic resonance imaging (MRI) and striatal dopaminergic deficit using single-photon emission computed tomography-dopamine transporter scan (SPECT-DaTSCAN)	Olgiati et al., 2015 PMID: 26077166

	CNV: Triplication	MLPA (detected), high-density array comparative genomic hybridization (aCGH) (detected dosage), qPCR (confirmed dosage)	Frontotemporal and parietal degeneration using FDG-positron emission tomography (PET), cortical and subcortical atrophia in the temporoinsular and the frontal regions using MRI, and putamen degeneration using SPECT-DaTSCAN	Ferese et al., 2015 PMID: 26635992
	CNV: Duplication	Semiquantitative PCR (detected), FISH (confirmed)	NA	Chartier-Harlin et al., 2004 PMID: 15451224
	CNV: Duplication	Semiquantitative multiplex PCR (detected)	NA	Ibáñez et al., 2004 PMID: 15451225
	CNV: Duplication	Semiquantitative PCR (detected), FISH (confirmed)	NA	Nishioka et al., 2006 PMID: 16358335
	CNV: Duplication	Semiquantitative multiplex PCR (detected), FISH (confirmed)	Decreased uptake of [123I] FP-CIT detected by [123I] FP-CIT SPECT	Ahn et al., 2008 PMID: 17625105
	CNV: Duplication	qPCR (detected), Affymetrix 250k SNP array (confirmed)	Reduction of dopamine transporters in the anterior and posterior putamen and caudate using [11C]-CFT PET, reduction in dopamine D2 receptor density in the caudate using [11C]-RAC PET, reduction in glucose metabolism in the occipital lobe using FDG-PET, and olfactory dysfunction using the "Sniffin' Sticks" assessment	Nishioka et al., 2009 PMID: 19562770
	CNV: Duplication	MLPA (detected), aCGH (copy number gain), SNP array (copy number gain homozygous), FISH (duplication on both alleles)	Reduction in tracer uptake in the striatum using DaTSCAN	Kojovic et al., 2012 PMID: 23208740

	CNV: Duplication	Next-generation sequencing (NGS) (detected)	Severe neuronal loss in the substantia nigra, the locus coeruleus, the dorsal motor nucleus of the vagus, the basal nucleus of Meynert, the amygdala, and the hippocampal CA2/3 region	Konno et al., 2015 PMID: 26350119
PRKN	Deletion	PCR (detected), southern blot (confirmed), reverse transcriptase PCR (confirmed)	NA	Kitada et al., 1998 PMID: 9560156
	Deletion	PCR (detected)	NA	Lucking et al., 1998 PMID: 9802278
	Deletion	PCR (detected)	NA	Abbas et al., 1999 PMID: 10072423
	Deletion, CNV: Duplication, Triplication	Semiquantitative PCR (detected)	NA	Lucking et al., 2000 PMID: 10824074
	Deletion	PCR (detected), dye terminator cycle sequencing (confirmed)	Neuronal loss in the substantia nigra pars compacta and no Lewy bodies	Hayashi et al., 2000 PMID: 11009195
	Deletion, CNV: Duplication	Quantitative duplex PCR (detected), southern blot (confirmed)	NA	Hedrich et al., 2001 PMID: 11487568
	Deletion, CNV: Duplication	Quantitative duplex PCR (detected)	NA	Hedrich et al., 2002 PMID: 11971093
	Deletion	Dye terminator cycle sequencing (detected), qPCR (confirmed)	NA	Nichols et al., 2002 PMID: 12114481
	Deletion, CNV: Duplication	Dye terminator cycle sequencing (detected), qPCR (confirmed)	NA	Foroud et al., 2003 PMID: 12629236
	Deletion, CNV: Duplication	Dye terminator cycle sequencing (detected), qPCR (confirmed)	NA	Poorkaj et al., 2005

				PMID: 15725358
	Deletion	Single-strand conformation polymorphism analysis (detected), quantitative duplex PCR (confirmed)	NA	Hedrich et al., 2004b PMID: 15390068
	Deletion	PCR (detected)	Neuronal degeneration in the substantia nigra and the locus coeruleus, and alpha-synuclein and ubiquitin-positive inclusions in the neuropils of the pedunculopontine nucleus	Sasaki et al., 2004 PMID: 15326242
	Deletion, CNV: Duplication	Denaturing high performance liquid chromatography (DHPLC) (detected), semiquantitative multiplex PCR (confirmed)	NA	Lesage et al., 2008 PMID: 17766365
	Deletion, CNV: Multiplication	MLPA (detected), qPCR (confirmed)	NA	Kay et al., 2010 PMID: 20876472
	Deletion, CNV: Duplication	Dye terminator cycle sequencing (detected), MLPA (confirmed)	NA	Kilarski et al., 2012 PMID: 22956510
	Deletion	Not specified	Neuronal loss in the substantia nigra and the locus coeruleus, and Lewy bodies found in the substantia nigra, locus coeruleus, dorsal motor nucleus of the vagus, the basal nucleus of Meynert, the amygdaloid nucleus, and the sympathetic nerve bundles in the myocardium	Miyakawa et al., 2013 PMID: 23401296
	Deletion	MLPA (detected), qPCR (confirmed)	NA	Taghavi et al., 2018 PMID: 28502045
	Deletion, CNV: Duplication	Sanger sequencing (detected), MLPA (confirmed)	NA	Milanowski et al. 2021

				PMID: 33845304
	Deletion	WGS (detected), MLPA (confirmed)	NA	Zhu et al., 2022 PMID: 35640906
	Deletion, CNV: Duplication	Sanger sequencing (detected), MLPA (confirmed)	NA	Yoshino et al., 2022 PMID: 35123805
	Deletion, Inversion	MLPA (detected), ONT long-read sequencing (confirmed)	NA	Daida et al., 2023 PMID: 37790330
PARK7	Deletion	Duplex PCR (detected), dye terminator cycle sequencing (confirmed)	NA	Bonifati et al., 2003 PMID: 14598065
	Deletion	Quantitative duplex PCR (detected)	NA	Hedrich et al., 2004a PMID: 14872018
	Deletion	DHPLC (detected), quantitative duplex PCR (confirmed)	NA	Djarmati et al., 2004 PMID: 15108293
	CNV: Duplication	Dye terminator cycle sequencing (detected), MLPA (confirmed)	NA	Macedo et al., 2009 PMID: 18973254
	Deletion	qPCR (detected)	NA	Guo et al., 2010 PMID: 20146068
	Deletion	MLPA (detected), qPCR (confirmed)	NA	Darvish et al., 2013 PMID: 23880019
	Deletion	NGS (detected), qPCR (confirmed)	NA	Spataro et al., 2017 PMID: 28124432
	Deletion	Whole exome sequencing (detected), MLPA (confirmed)	NA	Guler et al., 2021 PMID: 34605055
PINK1	Deletion	PCR (detected), dye terminator cycle sequencing (confirmed)	NA	Li et al., 2005 PMID: 15955953

	Deletion	Dye terminator cycle sequencing (detected), qPCR (confirmed)	NA	Marongiu et al., 2007 PMID: 17154281
	Deletion, Insertion, duplication	MLPA (detected), dye terminator cycle sequencing (confirmed)	NA	Cazeneuve et al., 2009 PMID: 19214605
	Deletion	MLPA (detected), qPCR (confirmed)	NA	Darvish et al., 2013 PMID: 23880019
	Deletion, CNV: Duplication	MLPA (detected), qPCR (confirmed)	NA	Monroy-Jaramillo et al., 2014 PMID: 24677602

Supplementary Table S1. SVs identified in PD genes *SNCA*, *PRKN*, *PARK7*, and *PINK1*.
